# Clinical implications of SPRR1A expression in diffuse large B-cell lymphomas: a prospective, observational study

**DOI:** 10.1186/1471-2407-14-333

**Published:** 2014-05-14

**Authors:** Hao Zhang, Jiyue Gao, Zuowei Zhao, Man Li, Caigang Liu

**Affiliations:** 1 Department of Breast Surgery, Second Hospital of Dalian Medical University, Dalian 116023, China; 2 Department of Oncology, Second Hospital of Dalian Medical University, Dalian 116023, China

**Keywords:** Diffuse large B-cell lymphomas, Survival, SPRR1A

## Abstract

**Background:**

Certain markers have been identified over the last 10 years that facilitate the prediction of a patient’s prognosis; these markers have been proposed to be useful for risk stratification of lymphoma patients and for the development of specific therapeutic strategies. In the present study, we assessed the potential prognostic value of SPRR1A expression in 967 patients with diffuse large B-cell lymphomas.

**Methods:**

All patients were enrolled between 2001 and 2007 (median follow-up, 53.3 months) in the Second Hospital of Dalian Medical University, First Hospital of China Medical University, and Liaoning Cancer Hospital. Immunohistochemical analysis was used to evaluate the expression of SPRR1A. Survival was analyzed using the Kaplan–Meier method. Multivariate analysis was conducted to adjust the effect of SPRR1A expression for potential, well-known, independent prognostic factors.

**Results:**

Of the 967 patients examined, SPRR1A expression was detected in 305 (31.54%) patients on immunohistochemical analysis. The 5-year survival rate was significantly lower in patients with SPRR1A expression than in those without (26.9% vs. 53.2%, *P* < 0.001). Multivariate analysis identified SPRR1A expression as an independent predictor of survival in addition to lactate dehydrogenase level, clinical stage, and histologic subtype.

**Conclusions:**

SPRR1A expression may be useful as a prognostic factor for diffuse large B-cell lymphoma.

## Background

Diffuse large B-cell lymphomas (DLBCL) constitute a heterogeneous category of aggressive lymphomas [1] that are diagnosed based on the morphology and immunophenotype [2,3], and represent 30–40% of cases of adult non-Hodgkin’s lymphoma [4]. Although the use of combination chemotherapy has improved the outcomes of DLBCL, many patients do not achieve complete remission (CR) and ultimately relapse. Therefore, it is important to determine factors that can assist with the identification of patients at a high risk of recurrent disease [5].

Immunohistochemical tests are routine procedures for the diagnosis of several malignancies and are considered to be essential in cases of lymphoma. Certain markers have been identified over the last 10 years that facilitate the prediction of a patient’s prognosis. These markers have been proposed to be useful for risk stratification of lymphoma patients and for the development of specific therapeutic strategies. Molecular abnormalities of the cell death–cell viability balance, as reflected in *bcl*-2 overexpression [6-9] or p53 mutation [10,11], have emerged as important prognostic indicators of DLBCL.

Small proline-rich (SPRR) proteins are characterized by an unusually high content of proline residues and were originally identified in cultured keratinocytes as ultraviolet-inducible genes [12,13]. Several studies have suggested that SPRRs are related to increased epithelial proliferation and malignant processes and are markers for terminal squamous cell differentiation, although they also function in nonsquamous tissues [14]. Moreover, primary basal cell carcinomas, squamous cell carcinomas, and thin melanomas have been reported to exhibit a considerably higher level of *SPRR1A* gene expression [15].

In the present study, we examined 967 specimens obtained from patients with DLBCL to investigate SPRR1A expression and its prognostic value.

## Methods

### Patient selection

The present study included patients (n = 2456) with a pathologically confirmed DLBCL diagnosis who were treated in the First Hospital of China Medical University, Fourth Hospital of China Medical University, or Liaoning Province Cancer Hospital between January 1, 2001, and December 31, 2007. At the time of the analysis, 39% of the slides were available for pathologic review, and 967 patients were considered to have DLBCL (centroblastic, immunoblastic, or anaplastic).

Disease dissemination was evaluated before treatment by physical examination, bone marrow (BM) biopsy, and computed tomography of the chest and abdomen. Patients were staged according to the Ann Arbor system. The number of extranodal sites and larger tumor mass diameters were also determined. Performance status was assessed according to the Eastern Cooperative Oncology Group scale: 0, patient had no symptoms; 1, patient had symptoms but was ambulatory; 2, patient was bedridden for less than half of the day; 3, patient was bedridden for half of the day or longer; and 4, patient was chronically bedridden and required assistance with activities of daily living. Performance status was then classified as 0–1 (the patient was ambulatory) or 2–4 (the patient was not ambulatory).

All the patients provided written informed consent, and the study protocol and the sample collection were approved by the Ethics Committee of China Medical University.

### Assessment of response

The primary endpoint was overall survival. Response to therapy was evaluated after the initiation of treatment. CR was defined as the disappearance of all clinical evidence of disease and normalization of all laboratory values, radiographs, computed tomography scans, and BM biopsy findings.

### Histologic and immunophenotypic study

The histologic diagnosis of DLBCL was independently determined by 3 pathologists. The diagnosis was based on morphologic examination of slides from routinely processed paraffin-embedded samples stained with hematoxylin-eosin, Giemsa, and Gordon–Sweet stains and on immunophenotyping results. The immunohistochemistry panel consisted of antibodies against CD20, CD10, CD3, CD5, BCL2, BCL6, IRF4/MUM1, human leukocyte antigen (HLA)–DR, and Ki-67.

Staining for CD20, CD3, CD10, and HLA-DR was scored as positive or negative. Each individual case was unanimously scored as negative or positive by the 3 independent investigators, scored using the 2 matching scores when the third investigator did not agree, or recorded as “not evaluable” for a given antigen when there was no agreement between the investigators.

Staining for CD5, BCL2, IRF4/MUM1, and BCL6 was scored in a semiquantitative manner, from 1 to 5, indicating the percentage of positive tumor cells: 1, no staining; 2, 5%–25%; 3, 26%–50%; 4, 51%–75%; and 5, >75%. For Ki-67 (MIB1), a score of 5 was defined as 76%–90%, and an additional score of 6 was introduced (>90%). Whenever individual cores for a given case showed nonconcordant results, the score of the core with the highest number of positive cells was recorded.

### Patient selection for SPRR1A expression

SPRR1A expression in DLBCL was analyzed when there were 4 unstained slides available for that case. Only formalin-fixed specimens were selected, while specimens fixed in Bouin’s fluid were excluded. To avoid bias related to treatment, only patients treated with CHOP were included in the study. Overall, 967 cases were studied for SPRR1A expression by immunohistochemical analysis.

### Immunohistochemical analysis

Thin slices of tumor tissue for all cases were fixed in 4% formaldehyde solution (pH 7.0) for a duration that did not exceed 24 hours. The tissues were processed in a routine manner for paraffin embedding, and 4-μm thick sections were cut and placed on glass slides coated with 3-aminopropyl triethoxysilane for immunohistochemical analysis. The sections were mounted on microscope slides, air dried, and then fixed in a mixture of 50% acetone and 50% methanol. The sections were then de-waxed with xylene, gradually hydrated with gradient alcohol, and washed with phosphate-buffered saline (PBS). Sections were then incubated for 60 minutes with the primary antibody. After repeated washing with PBS, the sections were incubated for 30 minutes with the secondary biotinylated antibody (Multilink Swine anti-goat/mouse/rabbit immunoglobulin; Dako, Inc.). Thereafter, the avidin-biotin complex (1:1000 dilution; Vector Laboratories, Ltd.) was applied to the sections for 30–60 minutes at room temperature. The immunoreactive products were visualized by catalysis of 3,3′-diaminobenzidine with horseradish peroxidase in the presence of H_2_O_2_, after extensive washing. Sections were then counterstained in Gill’s Hematoxylin and dehydrated in ascending grades of methanol, prior to clearing with xylene and mounting under a coverslip.

To identify immunopositive staining for SPRR1A, SPRR1A expression was first classified semiquantitatively according to the following criteria: 0, <1% of cells discretely expressed SPRR1A; 1+, ≥1 and <10% of cells discretely expressed SPRR1A; and, 2+, ≥10% of cells discretely expressed SPRR1A. Samples scored as 1+ or 2+ were considered positive.

### Statistical analysis

Patient characteristics were compared using the Chi-square test. Overall survival was analyzed using the Kaplan–Meier method. The log-rank test was used to analyze survival differences. Multivariate analysis was conducted to adjust the effect of SPRR1A expression for potential independent prognostic factors (age, sex, extranodal sites, performance status, clinical stage, bulky disease [_____ >10 cm], evolution, lactate dehydrogenase level, and SPRR1A expression) using the Cox proportional hazards model with forward stepwise selection. A *P* value of <0.05 was considered statistically significant. All data were analyzed using SPSS (Version 17.0; SPSS Inc., Chicago, IL, USA).

## Results and discussion

### SPRR1A expression in diffuse large B-cell lymphomas

**Figure 1:**
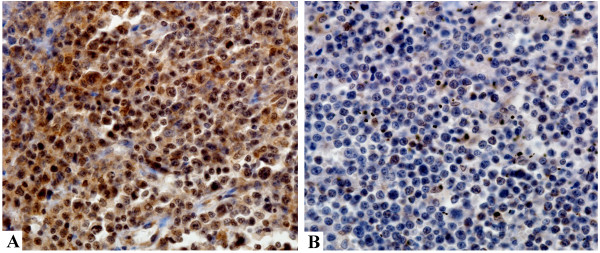
**Expression of SPRR1A in diffuse large B-cell lymphomas by immunohistochemical analysis. (A)** Representative case of large B-cell lymphoma that was positive for SPRR1A expression. **(B)** Representative case of large B-cell lymphoma that was negative for SPRR1A expression.

The median age of the population was 56 years. Except for histologic subtype, the clinical characteristics were well balanced between the 2 groups (SPRR1A − and SPRR1A + groups) (Table 1). We found that the lymphoma tissue was positive for anti-SPRR1A staining in 305 (31.54%) cases (Figure 1).

**Table 1:** Characteristics of patients with diffuse large B-cell lymphoma according to SPRR1A status

**Characteristics**	**SPRR1A− (n = 662)**	**SPRR1A+ (n = 305)**	***P *** **value**
**Age (years)**			0.712
Median	57	55	
**Sex (%)**			0.708
Men	483 (73)	219 (72)	
Women	179 (27)	86 (28)	
**Extranodal sites (%)**			0.203
0–1 site	480 (73)	209 (69)	
More than 1 site	182 (27)	96 (31)	
**Performance status (%)**			0.354
0–1	503 (76)	240 (79)	
2–4	159 (24)	65 (21)	
**Clinical stage (%)**			0.074
I or II	347 (52)	141 (46)	
III or IV	315 (48)	164 (54)	
**Bulky disease >10 cm (%)**			0.858
No	334 (51)	152 (49)	
Yes	328 (49)	153 (51)	
**IPI Score (%)**			0.218
0–1	342 (52)	150 (49)	
2	135 (20)	55 (18)	
3	105 (16)	65 (21)	
4–5	80 (12)	35 (12)	
**Lactate dehydrogenase (%)**			0.757
Normal	273 (41)	129 (42)	
Elevated	389 (59)	176 (58)	
**Evolution (%)**			0.087
CR	418 (64)	175 (57)	
No CR	244 (36)	130 (43)	

IPI, International Prognostic Index; CR, complete remission.

### SPRR1A expression and survival

**Figure 2:**
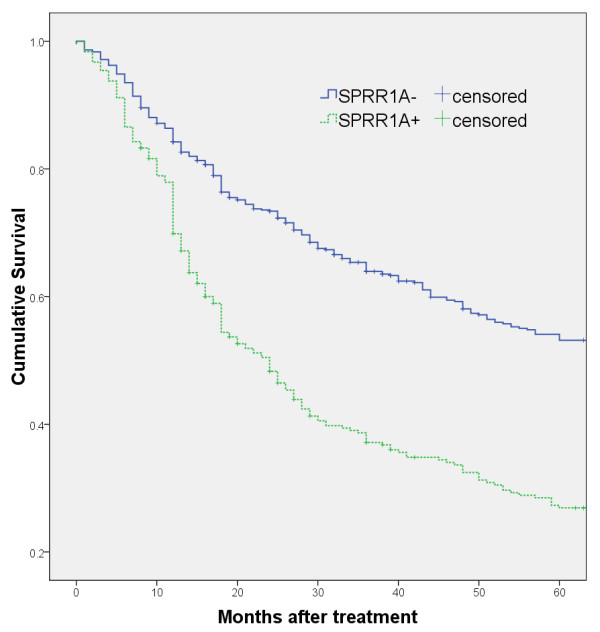
The 5-year survival curve of SPRR1A + and SPRR1A − patients with diffuse large B-cell lymphomas.

With a median follow-up of 53.3 months, the 5-year survival rate was significantly lower in the SPRR1A + group (26.9% [95% CI, 21.6–32.2%]) than in the SPRR1A − group (53.2% [95% CI, 48.9–57.5%]; *P* < 0.001) (Figure 2).

### Prognostic value of SPRR1A expression

The hazard ratio for death was 1.792 (95% CI, 1.364–3.778; *P* < 0.001) in the SPRR1A + group (Table 2, univariate analysis).

**Table 2:** Hazards ratios for death in an intention-to-treat population (n = 967) based on univariable and multivariable analyses

	**Univariable analysis**		**Multivariable analysis**	
	**HR (95% CI)**	***P*** *	**HR (95% CI)**	***P*** **†**
**Age (years)**		**0.033**		**0.421**
≤60 y	1 (Ref)		1 (Ref)	
>60 y	1.231 (1.017–1.490)	0.033	1.112 (0.762–1.397)	0.421
**Sex**		**0.816**		**0.736**
Women	1 (Ref)		1 (Ref)	
Men	1.025 (0.833–1.261)	0.816	1.103 (0.836–1.207)	0.736
**Extranodal sites**		**0.576**		**0.512**
0–1 site	1 (Ref)		1 (Ref)	
More than 1 site	1.080 (0.824–1.416)	0.576	1.375 (0.831–1.454)	0.512
**Performance status**		**0.058**		**0.062**
0–1	1 (Ref)		1 (Ref)	
2–4	1.755 (0.838–2.146)	0.058	1.172 (0834–1.884)	0.062
**Clinical stage**		**0.000**		**0.024**
I or II	1 (Ref)		1 (Ref)	
III or IV	1.742 (1.382–3.666)	0.000	1.217 (1.114–2.371)	0.024
**Bulky disease >10 cm**		**0.221**		**0.413**
No	1 (Ref)		1 (Ref)	
Yes	1.177 (0.591–1.529)	0.221	1.124 (0.734–1.349)	0.413
**Evolution**		**0.789**		**0.442**
CR	1 (Ref)		1 (Ref)	
No CR	0.901 (0.723–1.122)	0.789	0.834 (0.644–1.176)	0.442
**IPI Score**		**0.007**		
0–1	1 (Ref)			
2	1.236 (1.074–2.537)	0.003		
3	1.779 (1.362–2.778)	0.014		
4–5	2.343 (1.742–4.621)	0.000		
**Lactate dehydrogenase**		**0.011**		**0.031**
Normal	1 (Ref)		1 (Ref)	
Elevated	1.921 (1.347–3.312)	0.011	1.383 (1.152–2.511)	0.031
**SPRR1A expression**		**0.000**		**0.013**
Negative	1 (Ref)		1 (Ref)	
Positive	1.792 (1.364–3.778)	0.000	1.564 (1.337–2.464)	0.013

HR, hazard ratio; CI, confidence interval; Ref, reference category; IPI, International Prognostic Index; CR, complete remission.

*Derived from tests of HR for prognostic factors in the univariable model.

†Cox-regression analysis, controlling for all of the prognostic factors listed in the table, except IPI Score.

After adjustment for the 11 baseline variables with the use of Cox regression analysis, the hazard ratio remained similar (Table 2, multivariate analysis).

Expectedly, the multivariate analyses showed that clinical stage, lactate dehydrogenase level, and SPRR1A expression were independent prognostic factors (Table 2, multivariate analysis).

### Predictive value of the expression of SPRR1A for germinal center B-cell-like/non-germinal center B-cell-like diagnosis

Considering histologic diagnosis as the gold standard method for diagnosis, the sensitivity and specificity of SPRR1A − for the diagnosis of the subtype of germinal center B-cell-like (GCB)/non-GCB in 416 patients was 96.6% (197/204) and 95.8% (203/212), respectively. The positive predictive value and negative predictive value of SPRR1A − for GCB DLBCL was 95.6% (197/206) and 67.7% (203/300), respectively.

In the present study, we determined that SPRR1A expression is a predictive factor for overall survival with DLBCL. The classic prognostic factors for aggressive B-cell lymphomas (i.e., advanced clinical stage, unfavorable performance status, elevated lactate dehydrogenase values) and, consequently, the International Prognostic Index score were significantly associated with a poor outcome in these patients. Importantly, the multivariate analyses demonstrated that the influence of SPRR1A expression on overall survival was independent of these well-established prognostic factors. Moreover, SPRR1A can be used to determine the GCB and non-GCB subtypes of DLBCL.

## Conclusions

SPRR1A was identified as a potential new independent prognostic factor for DLBCL. These findings may provide a new molecular framework to identify patients harboring aggressive B-cell lymphomas.

## Abbreviations

BM: Bone marrow; CR: Complete remission; DLBCL: Diffuse large B-cell lymphoma; HLA: Human leukocyte antigen; SPRR: Small protein-rich; PBS: Phosphate buffered saline; GCB: Germinal center B-cell.

## Competing interests

The authors declare that they have no competing interests.

## Authors’ contributions

HZ carried out the experiment and drafted the manuscript. JG and CL participated in the design of the study and performed the statistical analysis. ZZ and ML conceived of the study, participated in its design and coordination, and helped to draft the manuscript. All authors read and approved the final manuscript.

## Pre-publication history

The pre-publication history for this paper can be accessed here:

http://www.biomedcentral.com/1471-2407/14/333/prepub
